# Arthroscopic near infrared spectroscopy enables simultaneous quantitative evaluation of articular cartilage and subchondral bone *in vivo*

**DOI:** 10.1038/s41598-018-31670-5

**Published:** 2018-09-07

**Authors:** Jaakko K. Sarin, Nikae C. R. te Moller, Irina A. D. Mancini, Harold Brommer, Jetze Visser, Jos Malda, P. René van Weeren, Isaac O. Afara, Juha Töyräs

**Affiliations:** 1 Department of Applied Physics, University of Eastern Finland, Kuopio, Finland; 2 Diagnostic Imaging Center, Kuopio University Hospital, Kuopio, Finland; 3 Department of Equine Sciences, Faculty of Veterinary Medicine, Utrecht University, Utrecht, The Netherlands; 4 Department of Orthopaedics, University Medical Center Utrecht, Utrecht, The Netherlands

## Abstract

Arthroscopic assessment of articular tissues is highly subjective and poorly reproducible. To ensure optimal patient care, quantitative techniques (*e.g*., near infrared spectroscopy (NIRS)) could substantially enhance arthroscopic diagnosis of initial signs of post-traumatic osteoarthritis (PTOA). Here, we demonstrate, for the first time, the potential of arthroscopic NIRS to simultaneously monitor progressive degeneration of cartilage and subchondral bone *in vivo* in Shetland ponies undergoing different experimental cartilage repair procedures. Osteochondral tissues adjacent to the repair sites were evaluated using an arthroscopic NIRS probe and significant (*p* < 0.05) degenerative changes were observed in the tissue properties when compared with tissues from healthy joints. Artificial neural networks (ANN) enabled reliable (*ρ* = 0.63–0.87, NMRSE = 8.5–17.2%, RPIQ = 1.93–3.03) estimation of articular cartilage biomechanical properties, subchondral bone plate thickness and bone mineral density (BMD), and subchondral trabecular bone thickness, bone volume fraction (BV), BMD, and structure model index (SMI) from *in vitro* spectral data. The trained ANNs also reliably predicted the properties of an independent *in vitro* test group (*ρ* = 0.54–0.91, NMRSE = 5.9–17.6%, RPIQ = 1.68*–*3.36). However, predictions based on arthroscopic NIR spectra were less reliable (*ρ* = 0.27–0.74, NMRSE = 14.5–24.0%, RPIQ = 1.35*–*1.70), possibly due to errors introduced during arthroscopic spectral acquisition. Adaptation of NIRS could address the limitations of conventional arthroscopy through quantitative assessment of lesion severity and extent, thereby enhancing detection of initial signs of PTOA. This would be of high clinical significance, for example, when conducting orthopaedic repair surgeries.

## Introduction

Osteoarthritis (OA) is a disabling disease associated with joint pain and restricted mobility, especially in the elderly^1,2^. Post-traumatic OA (PTOA), however, affects people of all ages and is initiated by joint trauma^2^, *e.g*., cartilage, meniscus, and ligament tears. These traumas conventionally require arthroscopic intervention, which is a common technique in both human and equine medicine. Mechanisms involved in the degeneration of articular cartilage have been extensively researched, and several studies^3,4^ have suggested changes in the subchondral bone properties to contribute to the initiation and progression of OA. In addition, subchondral bone is susceptible to morphological and compositional changes due to alterations in the stress distribution^5,6^; these changes can substantially influence joint functionality. Conventional arthroscopy is, however, based on qualitative visual and tactile assessment, rendering the technique subjective with suboptimal reliability^7,8^. Reliable evaluation of defects and the surrounding tissues is essential in choosing the optimal repair procedure and, thus, for halting the progression of PTOA^9,10^. This highlights the need for novel quantitative arthroscopic techniques, such as ultrasound^11^, optical coherence tomography (OCT^12^), and near infrared spectroscopy (NIRS^13^).

NIRS is a non-destructive optical technique in which the sample is irradiated with light, and the scattered and reflected light is collected. The technique enables rapid evaluation of tissues *in vivo* and eliminates the need for invasive, destructive, and slow chemical analysis^14^. NIRS enables swift assessment of cartilage biomechanical properties^13,15–18^ and composition^13^, as well as subchondral bone structure^19^. The spectral range between 0.4 and 2.5 µm enables assessment of the tissue at various depths due to the wavelength-dependent penetration depth^20^. The shorter wavelengths penetrate through the cartilage matrix into the subchondral and trabecular bone^21^, whereas longer wavelengths (closer to the mid infrared region) are restricted to the superficial layer of cartilage^20^. While there are several *in vitro* studies on the application of NIRS for cartilage assessment^13,15–18,21,22^, only a few have investigated the potential of this optical technique *in vivo*^23–27^. Furthermore, in these *in vivo* studies, cartilage condition was only evaluated using simple and poorly effective analytical approaches, such as determining the ratio of spectral peaks. Robust and effective analytical techniques, such as neural networks, could enable more accurate and reliable estimation of cartilage properties from the near infrared (NIR) spectral data.

Advances in multivariate analysis techniques and the increase in available computational resources have enabled accurate modelling of the relationships between complex NIR spectral data and reference parameters, such as cartilage biomechanical properties. Partial least squares regression (PLSR) is currently the most common technique in chemometrics for analysis of NIR spectral data^28^. However, artificial neural networks (ANN) combined with variable selection methods have recently been introduced for analysis of cartilage spectral data^22,29,30^ and have shown potential for evaluation of tissue properties.

We hypothesize that arthroscopic NIRS enables reliable simultaneous evaluation of articular cartilage and subchondral bone *in vivo* via adaptation of ANN. To test this hypothesis, arthroscopic and *in vitro* NIRS measurements were conducted on tissue surrounding experimental cartilage repair sites in equine joints at the 12-month end-point and compared with unaffected tissue harvested from matching sites in healthy control ponies. As references, articular cartilage biomechanical properties and subchondral bone microstructure and density were determined via indentation testing and computed tomography, respectively. To investigate the relationship between the spectral data and reference parameters, ANN with forward variable selection technique was adapted.

## Methods

Two cylindrical (*d* = 9 mm) chondral lesions were surgically created on the medial femoral ridges of both femoropatellar joints of Shetland ponies (*N* = 7, 6 females and 1 male, Age = 8.8 ± 3.5 years, total of 28 lesions). Each lesion was treated by filling it with Gelatin Methacryl (GelMA) hydrogel (3 varieties) or fibrin glue^31^. The repair procedure (*fibrin glue*, *GelMA cap*, *GelMA*, or *reinforced GelMA*) was randomized (proximal or distal lesion site and left or right knee) for each defect. A mixture of allogeneic mesenchymal stem cells (MSCs) and chondrons (80/20% ratio) at different concentrations was implanted in each defect. In the *fibrin glue* group, a low cell concentration (2 million cells/ml) was used, whereas the *GelMA cap* group had 1 million cells in a small volume of GelMA on the bottom of the defect, covered by a layer of cell free GelMA. In the last two groups, a high concentration (20 million cells/ml) was implanted in *GelMA* gel, and in the *reinforced GelMA* group, GelMA was reinforced with a 3D printed scaffold (melt electrospun polycarpolactone mesh)^32^.

After 12 months, the ponies were sacrificed and the osteochondral defects, together with the surrounding tissues, were examined via conventional and NIRS arthroscopes. Subsequently, osteochondral blocks were extracted for further analysis. As control, a similar osteochondral block was extracted from both femoropatellar joints of healthy ponies (*N*_*control*_ = 3, Age = 10.3 ± 4.7 years), ensuring an overall representative sample population. The animal studies were approved by the Ethics Committee of Utrecht University for Animal Experiments in compliance with the Institutional Guidelines on the Use of Laboratory Animals and carried out in a surgical theatre at the Department of Equine Sciences, Utrecht University, The Netherlands (Permission DEC 2014.III.11.098). The control ponies were acquired from a slaughterhouse in Utrecht, The Netherlands.

### Arthroscopic near infrared spectroscopy

**Figure 1:**
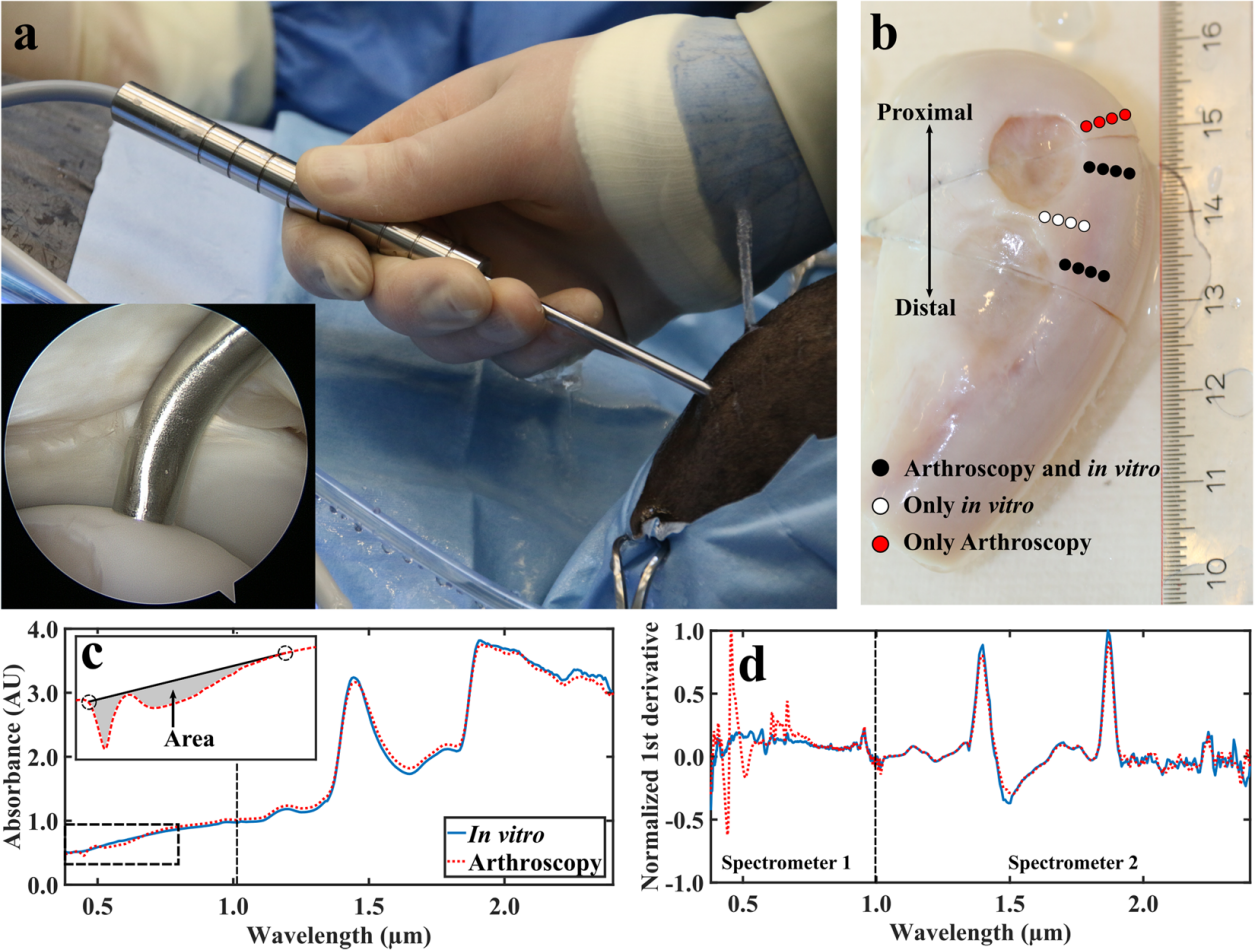
The novel fibre optic probe in an equine knee joint *in vivo* (**a**) with the probe tip in contact with cartilage surface (inset). Locations of NIRS measurements conducted *in vivo* during arthroscopy and *in vitro* in the laboratory (**b**). Comparison of average smoothed (**c**) and first derivative pre-processed (**d**, not used for modelling) spectra collected *in vivo* and *in vitro* with two separate spectrometers to cover the wide spectral region. NIRS measurement locations indicated with white and black dots were subjected to biomechanical and micro-CT reference measurements (**b**). For the red dots, values of reference parameters were only predicted based on ANN models. In subfigure (**c**), calculation of area between a two-point linear fit and measured spectrum was applied to detect outlier spectra. In subfigure (**d)**, the 1^st^ derivative spectra (not used for analysis) highlight the contribution of light from the conventional arthroscope at the spectral region of 0.42–0.75 µm.

Near infrared (NIR) spectral measurements (*N*_*ponies*_ = 7, *N*_*per joint*_ = 12, *N*_*total*_ = 164, 4 locations arthroscopically unreachable) were acquired arthroscopically *in vivo* by an experienced board-certified equine surgeon (>500 arthroscopies, Diplomate European College of Veterinary Surgeons) at the 12-month time-point immediately upon sacrifice. The arthroscopies were performed by utilizing a traditional arthroscope (4 mm, 30**°** inclination, Synergy HD3, Arthrex, Naples, FL, USA) as a monitoring tool and a novel, robust, and reusable arthroscopic NIRS fibre probe as a measurement tool (Fig. 1a). Twelve locations surrounding cartilage repair sites were measured (Fig. 1b) by orientating the fibre probe in perpendicular contact with cartilage surface. At each measurement point, 15 spectra were recorded, each being the average of ten successive spectra; the total duration of data acquisition was 2.4 seconds per measurement location. Arthroscopic images were recorded with a conventional arthroscope during the operation to enable reliable location tracking. Ringer’s solution (Fresenius, Bad Homburg v.d.H., Germany) containing sodium chloride (8.6 g/L), potassium chloride (0.3 g/L), and calcium chloride (0.33 g/L) was used for joint distension. Following the measurements, osteochondral samples were extracted after removing the skin and overlying tissues of the joint (Fig. 1b). The samples were frozen (−20 °C) until required for laboratory NIRS, biomechanical, and computed tomography (CT) measurements.

### *In vitro* near infrared spectroscopy

The *in vitro* NIRS measurements (*N*_*ponies*_ = 10, *N*_*per joint*_ = 12, *N*_*total*_ = 236; same 4 locations excluded as during arthroscopy) were acquired in similar conditions as in arthroscopy, *i.e*., hardware, immersion solution, and temperature, apart from using the conventional arthroscope for navigation. At each measurement point, three successive spectra were acquired, each consisting of 10 co-added scans.

The NIRS system consisted of spectrometers (AvaSpec-ULS2048L, λ = 0.35–1.1 µm, resolution = 0.6 nm and AvaSpec-NIR256-2.5-HSC, λ = 1.0–2.5 µm, resolution = 6.4 nm, Avantes BV, Apeldoorn, The Netherlands), a light source (AvaLight-HAL-(S)-Mini, λ = 0.36–2.5 µm, Avantes BV), and a custom arthroscopic fibre probe (Avantes BV). The reusable stainless-steel fibre probe (*d* = 3.25 mm) is sterilisable in an autoclave at 121 °C and its tip resembles the shape of a traditional arthroscopic hook. The probe tip window (*d* = 2 mm) contains 114 optical fibres (*d* = 100 µm), with 100 fibres emitting and 14 fibres (7 + 7) collecting light to the spectrometers. Avasoft software (version 8.7.0, Avantes BV) was used for spectral acquisition.

### Spectral preprocessing

A 3^rd^ order Savitzky-Golay filter was applied for smoothing of spectral data separately for the two spectrometers due to differences in their wavelength resolution. For cartilage and subchondral bone properties, the smoothing points were 25 and 13, and 45 and 13 points, respectively. The spectral region 1.9–2.5 µm was discarded from the analyses due to spectral saturation caused by high absorption of water.

In the arthroscopic NIRS measurements, interference from the light source of the conventional arthroscope was observed in the visible spectral region (λ ≈ 0.42–0.75 µm, Fig. 1d). Therefore, this region was applied only as an indicator for probe orientation and not used in the modelling. For arthroscopic NIRS, sufficient contact of the probe with cartilage surface is essential, as the irrigation fluid (*i.e*., saline) is an effective absorber of NIR light. To evaluate contact between the probe and cartilage surface, the area between a two-point linear fit and the measured spectrum (in a spectral region of 0.42–0.75 µm) was calculated (Fig. 1c). Four measurement locations were excluded due to high contribution from the arthroscope light source. Additionally, seven spectra (with the largest area between a linear fit and measured spectrum) out of the fifteen measured spectra from all measurement locations were excluded. Coefficient of variation was determined between the arthroscopic and *in vitro* spectra in spectral region 0.75–1.9 µm (3.7 ± 1.9%)^17^.

### Optical coherence tomography

**Figure 2:**
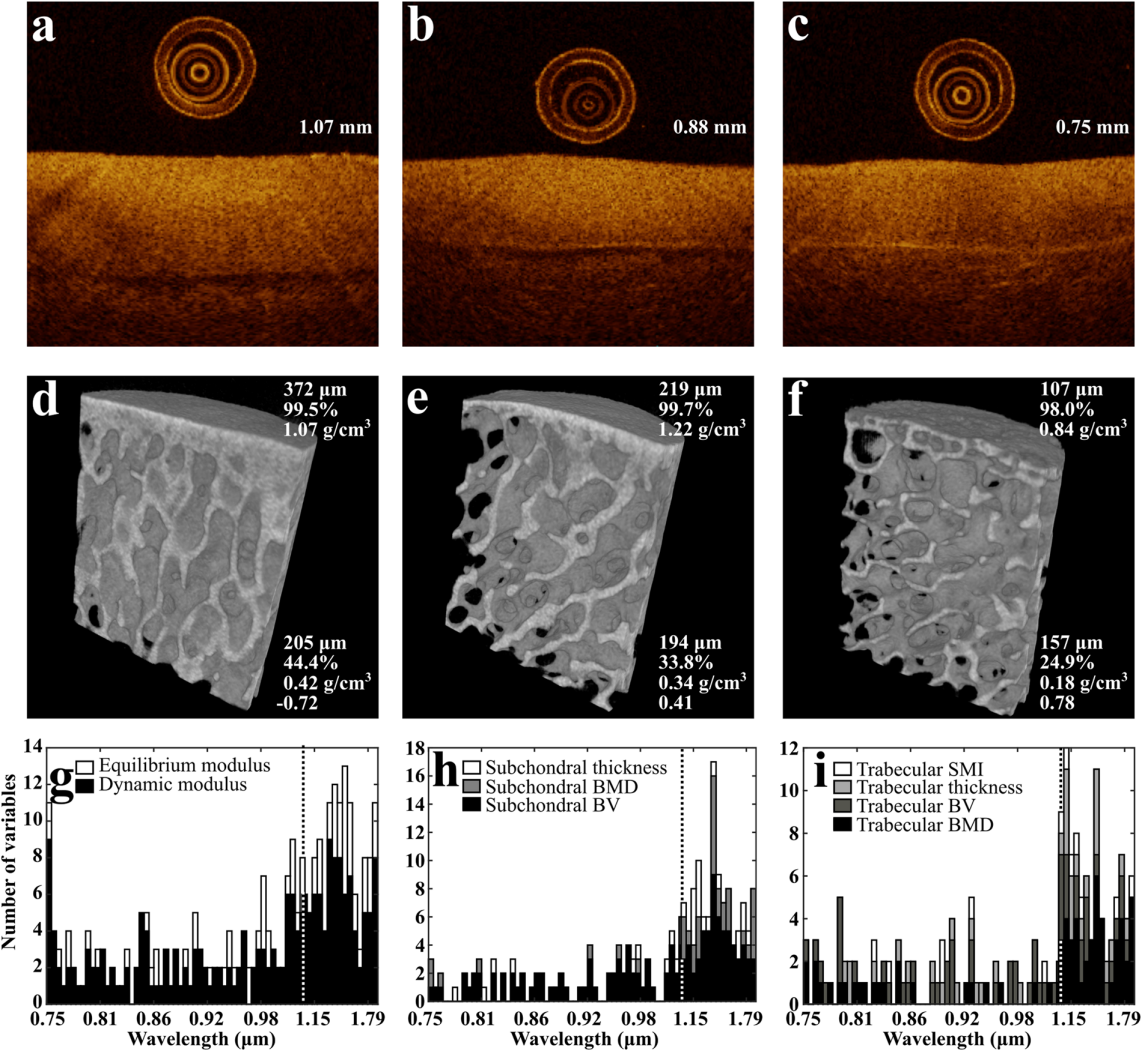
Optical coherence tomography (OCT) images from 3 locations, including cartilage thickness (**a**–**c**) along with corresponding micro-CT images of the underlying subchondral bone plate and subchondral trabecular bone (**d**–**f**). Subchondral plate thickness, bone volume fraction (BV), and bone mineral density (BMD) (**d**–**f**, top-right corner), and trabecular bone thickness, BV, BMD, and structural model index (SMI) (**d**–**f**, bottom-right corner) are presented. In addition, the optimal wavelengths for ANN models (Model 1) are presented (**g**–**i**). The width of each bar is 10 wavelengths (*i.e*., maximum number of variables in subfigure (**g**) is 20, as two variables are displayed). The dashed line indicates a separation of the spectral measurement regions of the two spectrometers.

After *in vitro* NIRS measurements, the measurement locations were marked with a felt tip pen. These points were imaged with OCT (λ = 1305 ± 55 nm, axial resolution < 20 µm, lateral resolution 25–60 µm; Ilumien PCI Optimization System, St. Jude Medical, St. Paul, MN, USA) by aligning a catheter (C7 Dragonfly, St. Jude Medical) over the measurement points (Fig. 1b) and performing a pullback imaging, thus imaging the NIRS measurement locations and the surrounding tissue (Fig. 2a–c). The samples were submerged in phosphate-buffered saline (PBS) during the imaging. Cartilage thickness was then determined from the OCT images of each location for biomechanical measurements.

### Biomechanical measurements

Biomechanical properties of cartilage surrounding the repairs (*N*_*ponies*_ = 10, *N*_*per joint*_ = 12, *N*_*total*_ = 236) were determined via indentation testing. The samples were glued on a custom-made sample holder, which was mounted on a goniometer (Model #55-841, Edmund Optics Inc., Barrington, NJ, USA) to align the cartilage surface perpendicular with the face of a plane-ended non-porous cylindrical indenter (*d* = 0.53 mm). Measurements were performed with the samples submerged in PBS. The indentation system consisted of a 250 g load cell (accuracy ± 0.25%, Model 31, Honeywell Sensotec Sensors, Columbus, OH, USA) and an actuator (displacement resolution 0.1 µm, PM500-1 A, Newport, Irvine, CA, USA).

First, the indenter was driven into initial contact with the sample. The contact was then ensured by indenting the sample five times using 2% strain. The measurement protocol consisted of four stress-relaxation steps (each of 5% strain) with a ramp velocity of 100%/s and a relaxation time of 600 seconds between the steps, followed by dynamic sinusoidal loading (*f* = 1.0 Hz) with a strain amplitude of 1%. The equilibrium modulus (*E*_*eq*_) was determined from the linear region of the stress-relaxation curve by assuming Poisson’s ratio of *ν* = 0.1, whereas the dynamic modulus (*E*_*dyn*_) was calculated as a ratio of the stress and strain amplitudes of the sinusoidal loading assuming a Poisson’s ratio of *ν* = 0.5^33,34^.

### Computed tomography and segmentation

Samples were imaged while submerged in PBS with a micro-CT scanner (Skyscan 1172, Skyscan, Kontich, Belgium) to determine subchondral bone plate and trabecular bone bone volume fraction (BV), bone mineral density (BMD), bone thickness, and trabecular bone structure model index (SMI). The samples were imaged using an isotropic voxel size of 12.15 × 12.15 × 12.15 µm^3^ and 100 kV tube voltage, along with hydroxyapatite phantoms (500, 1000, and 1250 mg/cm^3^). To ensure reliable location tracking for the segmentation, plastic cubes of approximately 8 mm^3^ were set on the NIRS measurement locations (Fig. 1b). From each measurement location, a cylindrical (diameter = 4.0 mm, height = 5.0 mm) region of interest (ROI) was virtually extracted, and the subchondral plate and trabecular bone were segmented (Fig. 2d–f). The extraction, segmentation, and analysis of bone properties were performed with DataViewer (Skyscan) and CTAn (Skyscan) programs. A global segmentation threshold (BMD = 0.46 g/cm^3^) was determined by comparing the binarized and original grayscale images.

### Artificial neural network

The relationship between NIR spectra and reference parameters was investigated using ANN. The ponies were divided into calibration (60%, *N* = 4 and *N*_*control*_ = 2, 142 spectra), validation (30%, *N* = 2 and *N*_*control*_ = 1, 70 spectra), and test (10%, *N* = 1, 24 spectra) groups. For each reference parameter, *in vitro* models with spectral regions 0.75–1.90 µm (Model 2) and 0.40–1.90 µm (Model 3) were developed based on *in vitro* NIR spectra and optimized by determining the most reliable wavelengths via the forward variable selection technique^22,29^. The optimal model was chosen based on the smallest root mean square error (RMSE) of the test group. Additionally, a model (0.75–1.90 µm, Model 1) was developed in which the most reliable wavelengths were determined by evaluating RMSE values of two sets: the test group and the arthroscopic measurements. For each location of the arthroscopic measurements, the final predicted value was resolved as an average of non-negative predicted values. During ANN modelling, the Levenberg-Marquardt backpropagation algorithm was used, while hyperbolic tangent and linear activation functions were employed in the hidden and output layers, respectively. To avoid overfitting, model training was halted after the validation error did not decrease in six successive iterations. The neural network architecture was limited to a maximum of eight neurons in the hidden layer and the analysis was performed in MATLAB (Matlab R2017b, MathWorks Inc., Natick, MA, USA) using the neural network toolbox (Version 11.0). To further evaluate model performance and reliability, the normalized RMSE (NRMSE) and ratio of performance to inter-quartile range (RPIQ)^35^ were calculated for *in vitro* training set (calibration and validation), independent test group, and arthroscopic predictions separately. The NRMSE is determined as RMSE relative to the reference parameter range, whereas RPIQ is determined as

1 $${\rm{RPIQ}}=\,\frac{{\rm{IQR}}}{{\rm{RMSE}}},$$

where IQR is the inter-quartile range of the measured data. The RPIQ was chosen due its suitability for non-normally distributed data^35^ and the threshold for reliable models was set to RPIQ ≥ 2, based on previous studies^36,37^.

### Statistical analyses

Reference properties had a non-normal distribution (Shapiro-Wilk normality test, *p* < 0.0003) and, thus, non-parametric tests were employed in statistical analysis. Statistical significance of differences in tissue properties between cartilage repair and control ponies was investigated by using the Mann-Whitney U test in SPSS (Version 23, SPSS Inc., IBM Company, Armonk, NY, USA); *p* < 0.05 was set as the limit for statistical significance. Two-tailed Spearman (*ρ*) correlation coefficients were determined between the measured and NIRS predicted reference parameter values.

Data of the current study is available from the corresponding author on reasonable request.

## Results

**Figure 3:**
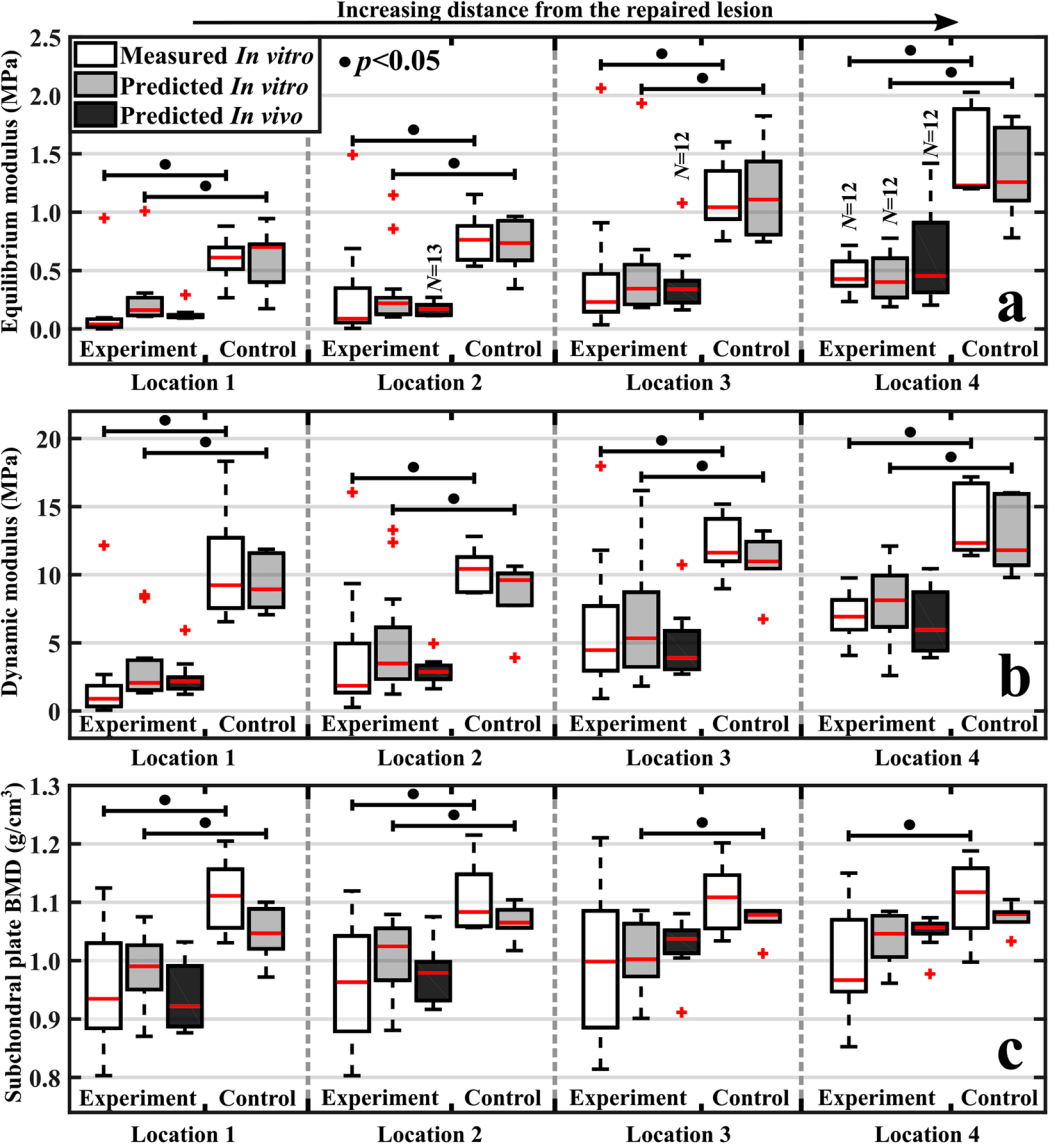
Boxplots for experiment (post-traumatic) and control (healthy) groups with median (red line), quartiles (25% and 75%), and outliers (red cross) of *in vitro* measured (white bars) cartilage equilibrium modulus (**a**), dynamic modulus (**b**), and subchondral bone plate BMD (**c**) for the four locations at increasing distances from the lesion. Additionally, predictions based on the optimal model (Model 1) for *in vitro* and arthroscopic NIRS measurements are presented (grey and black bars, respectively). For each location, experiment and control groups had 12–14 and 6 measurements, respectively.

Significant differences (*p* < 0.05) between the cartilage repair and control groups were observed in values of measured cartilage biomechanical properties (adjacent to the repair site), subchondral bone plate BV, and BMD (Figs. 2, 3, and Supplementary Figs. S1–5). Nevertheless, no significant differences (*p* = 0.16–0.93) were observed in cartilage thickness between the groups (Supplementary Fig. S1). The optimal predictive models (Model 1, *ρ*_Calibration&Validation_ = 0.63–0.87, RPIQ_Calibration&Validation_ = 1.93–3.03) reliably predicted the tissue properties (*ρ*_Test_ = 0.54–0.91, RPIQ_Test_ = 1.68–3.36) of the independent test group apart from subchondral bone BV (*ρ*_Calibration&Validation_ = 0.69, RPIQ_Calibration&Validation_ = 1.34, *ρ*_Test_ = 0.58, RPIQ_Test_ = 1.30) from *in vitro* spectral data (Table 1) for the four locations at different distances from the repaired lesion (Fig. 3), thus accurately differentiating between healthy and post-traumatic tissue. Furthermore, cartilage models (average RPIQ_Calibration&Validation_ = 2.76, RPIQ_Test_ = 3.26) were superior to subchondral bone plate (average RPIQ_Calibration&Validation_ = 1.99, RPIQ_Test_ = 1.95) and trabecular bone models (average RPIQ_Calibration&Validation_ = 2.09, RPIQ_Test_ = 2.27). In addition, arthroscopic predictions with optimal predictive models (Model 1) also enabled differentiation between healthy and post-traumatic tissue (Fig. 3). Although, the prediction performance (*ρ* = 0.27–0.74, RPIQ = 0.81–1.70) was inferior when compared to *in vitro* predictions, possibly due to errors (*e.g*., non-perfect contact of the probe and cartilage surface) introduced during arthroscopic spectral acquisition.

**Table 1:** Two-tailed Spearman (*ρ*) correlation coefficients between the measured and predicted values of cartilage: equilibrium (*E*_*eq*_) and dynamic moduli (E_*dyn*_), subchondral bone plate: bone volume fraction (BV), bone mineral density (BMD), and thickness, and trabecular bone: BV, BMD, thickness, and structure model index (SMI).

		Mean (95% Confidence interval)	Spectral region (µm)	Number of variables	*In vitro*						Arthroscopic			
					*Calibration and Validation*			*Test*						
					*ρ*	NRMSE	RPIQ	*ρ*	NRMSE	RPIQ	*ρ*	*p*	NRMSE	RPIQ
Cartilage	*E*_*eq*_ (kPa)	579 (506, 652)	0.75–1.90*	74	0.873	8.5%	3.14	0.890	5.9%	3.36	0.736	<0.0001	14.5%	1.46
			0.75–1.90	85	0.874	9.0%	2.96	0.835	7.0%	2.85	0.466	<0.0001	23.5%	0.90
			0.40–1.90	52	0.699	17.6%	1.51	0.876	7.2%	2.76	—	—	—	—
	*E*_*dyn*_ (Mpa)	7.25 (6.61, 7.89)	0.75–1.90*	214	0.775	16.5%	2.38	0.910	14.0%	3.16	0.692	<0.0001	19.7%	1.70
			0.75–1.90	61	0.793	15.8%	2.50	0.943	12.2%	3.63	0.544	<0.0001	21.8%	1.53
			0.40–1.90	133	0.853	12.4%	3.19	0.963	7.3%	6.07	—	—	—	—
Subchondral bone plate	BV (%)	98.4 (98.1, 98.7)	0.75–1.90*	133	0.689	7.1%	1.34	0.582	23.2%	1.30	0.384	<0.0001	10.4%	0.81
			0.75–1.90	171	0.790	4.7%	2.03	0.867	9.5%	3.17	0.358	0.0002	18.5%	0.46
			0.40–1.90	110	0.737	3.8%	2.45	0.697	10.3%	2.93	—	—	—	—
	BMD (g/cm^3^)	1.00 (0.99, 1.02)	0.75–1.90*	39	0.632	17.2%	2.18	0.539	17.6%	1.68	0.268	0.0063	24.0%	1.61
			0.75–1.90	88	0.725	15.0%	2.49	0.732	16.0%	1.85	−0.179	0.07	31.3%	1.23
			0.40–1.90	18	0.544	18.3%	2.04	0.757	15.6%	1.90	—	—	—	—
	Thick. (µm)	174 (166, 182)	0.75–1.90*	27	0.729	14.0%	2.45	0.896	9.9%	2.86	0.507	<0.0001	22.0%	1.45
			0.75–1.90	182	0.750	18.9%	1.81	0.950	12.0%	2.36	0.364	0.0002	32.8%	0.97
			0.40–1.90	143	0.794	18.5%	1.85	0.896	12.9%	2.19	—	—	—	—
Trabecular bone	BV (%)	30.5 (29.8, 31.3)	0.75–1.90*	56	0.781	11.2%	2.26	0.871	12.7%	2.15	0.547	<0.0001	20.8%	1.46
			0.75–1.90	28	0.703	12.4%	2.04	0.911	10.5%	2.60	0.325	0.0008	32.3%	0.94
			0.40–1.90	39	0.742	12.3%	2.06	0.949	10.6%	2.57	—	—	—	—
	BMD (g/cm^3^)	0.247 (0.237, 0.258)	0.75–1.90*	69	0.735	13.2%	2.08	0.856	14.7%	1.89	0.346	0.0003	22.9%	1.35
			0.75–1.90	103	0.739	13.3%	2.06	0.795	16.0%	1.73	0.266	0.0066	25.4%	1.22
			0.40–1.90	36	0.763	11.8%	2.30	0.929	11.6%	2.40	—	—	—	—
	Thick. (µm)	169 (167, 172)	0.75–1.90*	34	0.679	14.4%	1.86	0.816	16.9%	2.46	0.442	<0.0001	17.6%	1.50
			0.75–1.90	69	0.776	12.4%	2.16	0.850	13.3%	3.12	−0.065	0.51	25.7%	1.03
			0.40–1.90	63	0.628	14.8%	1.81	0.821	14.0%	2.98	—	—	—	—
	SMI	0.367 (0.316, 0.417)	0.75–1.90*	13	0.728	11.8%	2.15	0.889	12.9%	2.59	0.465	<0.0001	19.8%	1.64
			0.75–1.90	104	0.844	10.6%	2.38	0.906	11.1%	3.02	0.217	0.028	37.0%	0.88
			0.40–1.90	52	0.825	10.9%	2.33	0.886	9.9%	3.40	—	—	—	—

*Calibration and Validation* indicates the correlation coefficient for both calibration and validation groups (nine ponies, *N * = 212), whereas the *Test* indicates the correlation coefficient for the independent test group (one pony, *N* = 24). In addition, the normalized root mean square error (NRMSE) and ratio of performance to inter-quartile range (RPIQ) are presented for these groups as well as for the arthroscopic data. The upmost row (*) of each parameter, indicates the model with optimized wavelength selection for arthroscopic predictions (Model 1). For all *in vitro* correlations, *p-*values were <0.01.

For the locations with only arthroscopic measurements (Methods: Fig. 1b, red dots), the trends of predicted values (Model 1) were consistent with those of measured values at other locations (Supplementary Figs. S1–5). Additionally, predictive models based on *in vitro* spectral data from the 0.75 to 1.9 µm region (Model 2) were optimal for estimating cartilage and subchondral bone plate properties, whereas models with a wider spectral region (0.4–1.9 µm, Model 3) incorporating the visible region were optimal for predicting subchondral trabecular bone properties (Table 1).

## Discussion

Currently, no quantitative arthroscopic tools are available for evaluation of cartilage and subchondral bone and thus orthopaedic surgeons have to manage with subjective visual scoring of injury severity and tissue probing with a metallic hook^7^. Although several arthroscopic instruments for biomechanical assessment of cartilage (*e.g*., Artscan^38^) have been introduced, several practical issues (*e.g*., poor inter-observer reliability) have limited their usage during routine arthroscopies^38^. In addition, ultrasound and OCT imaging have been suggested, but these have not yet gained wide acceptance for arthroscopic evaluation of cartilage^11,12^. For arthroscopic evaluation of subchondral bone, there are currently no clinical tools available; nevertheless, arthroscopic ultrasound imaging has been shown to provide information on the subchondral bone^39^. CT and MRI are widely used for diagnostics of joint injuries, but these techniques cannot be utilized during arthroscopic repair surgery. As a result, the presently introduced NIRS probe could be used to accurately localize cartilage and bone defects, as well as the spread of tissue degeneration from an injury during arthroscopy, therefore potentially leading to better outcome of the tissue repair. Predictive models based on ANN provided accurate estimates of reference parameters for an independent *in vitro* test group. Although predictions based on spectra collected during *in vivo* arthroscopies had slightly higher errors (*i.e*., weaker performance), they were able to discriminate between healthy and post-traumatic tissue. These findings suggest that NIRS is a promising technique for *in vivo* assessment of articular cartilage and subchondral bone properties.

Near infrared spectroscopy has been applied previously in human arthroscopies by Spahn *et al*. and Hofmann *et al*. to evaluate the condition of cartilage^23–27^. However, no study has applied NIRS for evaluation of subchondral bone properties *in vivo*, or for simultaneous assessment of cartilage and subchondral bone integrity, or for prediction of tissue properties based on *in vivo* NIRS arthroscopy. In the aforementioned studies, a spectral region of 0.9–1.7 µm was utilized, whereas a wider region of 0.4–1.9 µm was utilized in the present study. Furthermore, the previous studies applied a simple univariate approach based on the ratio of spectral peaks for assessments of cartilage condition^25,27^, while a more advanced analytical approach based on ANN was adopted in this study.

The spectral region 0.75–1.9 µm was optimal for prediction of cartilage biomechanical and subchondral bone plate properties (*i.e*., subchondral plate thickness, BV, and BMD), while models that also incorporated the visible region (0.4–0.75 µm) enhanced the reliability of predicting subchondral trabecular bone properties (*i.e*., trabecular thickness, BV, BMD, and SMI). This is due to better penetration of visible light through cartilage and into subchondral bone^20^. Nevertheless, the errors of arthroscopic predictions were higher for subchondral bone plate and trabecular bone properties compared to cartilage biomechanical properties, possibly due to contributions from the overlying cartilage matrix. Furthermore, since probe contact with the cartilage surface affects the transmission of light into the tissue, this is possibly the reason for the weaker prediction of bone properties based on arthroscopic spectral measurements. Detailed understanding of wavelength-dependent light penetration would provide insight that enables quantification of the effect of the overlying cartilage in future studies.

The overall errors of prediction based on spectral data collected during arthroscopies were higher compared to those based on *in vitro* measurements. This is probably due to the difficulty in ensuring perfect probe contact with cartilage surface during arthroscopic spectral acquisition due to the geometrical constraints in the live situation. In the analysis, predictions based on the best 8 arthroscopic spectra (out of 15 recorded for each measurement location) resulted in the most reliable predictions; however, these spectra were not necessarily obtained through perfect probe contact. To enhance the identification of optimal spectra for each measurement location, additional indicators or classification algorithms, *e.g*., support vector machines and decision trees, could be utilized. However, this was beyond the scope of the current investigation. Additionally, for future studies the pressure between the arthroscopic NIR probe and cartilage surface should be quantitatively measured in order to minimize its effect on the resulting NIR spectra and consequently the prediction accuracy^40,41^.

The gold standard multivariate technique used in multiple applications (*e.g*., determining soil properties) is PLSR; however, ANN has in many occasions outperformed PLSR^42,43^. Furthermore, ANN modelling does not require extensive preprocessing^22^, whereas PLSR often requires scatter correction and derivative pre-processing for optimal performance. Generally, ANNs, in particular deep neural networks (≥2 hidden layers), are considered to require more data. However, only shallow neural networks (with single hidden layer) were utilized in this study. Therefore, similar estimates of minimum number of observations can be applied for shallow ANN models as multivariate models, such as PLSR and multiple linear regression (MLR). Consequently, we deem over hundred observations to be sufficient with more being always better. In addition, roughly 200 observations were recommended by Bujang *et al*. when applying MLR^44^. Although the number of spectra (*N* = 24) in the independent test set was low, this test provides an unbiased evaluation of model performance. Furthermore, the relatively small prediction errors ensured that the models were well-generalized for new samples. As expected, the forward variable selection technique improved the performance and robustness of the models by reducing the number of wavelengths; this is consistent with our previous study^22^. The prediction errors in cartilage biomechanical properties based on arthroscopic spectra were substantially lower with the variable selection technique employed in the present study compared to the genetic algorithm approach applied in our previous study^30^.

The correlations demonstrated by the models arise from overtone vibrations of chemical bonds in the main constituents of articular cartilage and subchondral bone, *i.e*., water, proteoglycans, collagen (types I and II), and hydroxyapatite. The most common bonds in these tissue constituents are OH, SH, NH, CH, and PO_4_^45,46^. Water is the most abundant constituent (up to 80%) in cartilage^45^ and thus the OH bond has a substantial influence on the spectral response of cartilage. The main peaks associated with the tissue water content appear between 0.95–1.10 µm, 1.40–1.55 µm, and 1.80–2.00 µm due to second overtone OH stretching, first overtone OH stretching, and second overtone OH bending vibrations, respectively^47^. The spectral regions 0.95–1.10 µm and 1.40–1.55 µm also include contributions from the second and first overtones of NH stretching vibrations^47^, respectively. In addition, overtones of CH stretching vibrations can be observed at spectral regions 0.85–0.95 µm, 1.10–1.25 µm, and 1.65–1.80 µm^47^. Spectral data from regions of stronger vibrations (first overtones) contribute more to model performance as observed from the histograms of selected wavelengths (Fig. 2g–i). The spectral region associated with the first overtone OH stretching vibration (1.40–1.55 µm) contributes substantially to the models, particularly for articular cartilage and subchondral bone plate parameters. For subchondral trabecular bone, the emphasis shifts towards the lower wavelengths (1.05–1.2 µm) due to better penetration depth of the light^20^.

The relationship between NIR spectra and cartilage biomechanical properties has been previously investigated *in vitro* with univariate^15,18^ and multivariate^13,16,17^ analysis (*i.e*., PLSR). These studies utilized relatively narrow spectral regions with moderate and good correlations, whereas in this study a wider region was utilized along with ANN combined with a variable selection technique. Afara *et al*. investigated subchondral bone properties with a similar spectral region in a rat model with promising results^19^. However, cartilage in a rat knee joint is substantially thinner than in human and equine knee joints^48,49^. Thus, the findings in the present study indicate that NIRS is a feasible technique for assessment of subchondral bone properties, even through thicker human and equine cartilage. Nevertheless, additional studies are required to confirm the validity of the NIRS technique in human patients.

No statistically significant difference was observed in cartilage thickness between control ponies and ponies with repaired cartilage defects. However, significant variation in cartilage biomechanical properties was observed between the groups. In PTOA, cartilage surrounding the site of a defect experiences higher strains^6^, therefore altering the tissue’s biomechanical competence and possibly leading to remodelling of the subchondral bone^50^. For subchondral plate properties, statistically significant differences were observed in values of BV and BMD between the groups, which is consistent with current knowledge on early-stage subchondral bone changes in OA^50,51^.

A possible limitation of this study is the potential effect of dependency as arthroscopic and *in vitro* measurements were conducted on the same ponies (the experimental group); however, this enabled comparison between the *in vitro* and *in vivo* environments, and provided valuable information for further development of *in vivo* NIRS applications. An additional limitation is that the arthroscopic spectra were found to include contributions from the conventional arthroscope light source, thereby limiting the useful spectral range to the NIR region (0.75–1.90 µm) because of interference in the visible spectral region.

### Outlook

Near infrared spectroscopy is a promising quantitative technique for simultaneous arthroscopic assessment of cartilage biomechanical properties and subchondral bone structure and density. This technique could substantially enhance assessment of the clinical status of joints by enabling quantitative detection of initial signs of PTOA around chondral lesions. This would be of high clinical significance, *e.g*., when conducting articular repair surgery.

## Electronic supplementary material

Supplementary information
