# Supplementary material for: Arthroscopic near infrared spectroscopy enables simultaneous quantitative evaluation of articular cartilage and subchondral bone in vivo
Source: Sci Rep. 2018 Sep 7;8:13409. doi: 10.1038/s41598-018-31670-5 (PMC6128946; doi:10.1038/s41598-018-31670-5)
Supplement: Supplementary file 1 — Supplementary information [file 41598_2018_31670_MOESM1_ESM.pdf]

Supplementary information:

Arthroscopic near infrared spectroscopy enables simultaneous quantitative evaluation of articular cartilage and subchondral bone *in vivo*

*Jaakko K Sarin [1,2]

Nikae CR te Moller [3]

Irina AD Mancini [3]

Harold Brommer [3]

Jetze Visser [4]

Jos Malda [3,4]

P René van Weeren [3]

Isaac O Afara [1]

Juha Töyräs [1,2]

[1] Department of Applied Physics, University of Eastern Finland, Kuopio, Finland

[2] Diagnostic Imaging Center, Kuopio University Hospital, Kuopio, Finland

[3] Department of Equine Sciences, Faculty of Veterinary Medicine, Utrecht University, Utrecht, The Netherlands

[4] Department of Orthopaedics, University Medical Center Utrecht, Utrecht, The Netherlands

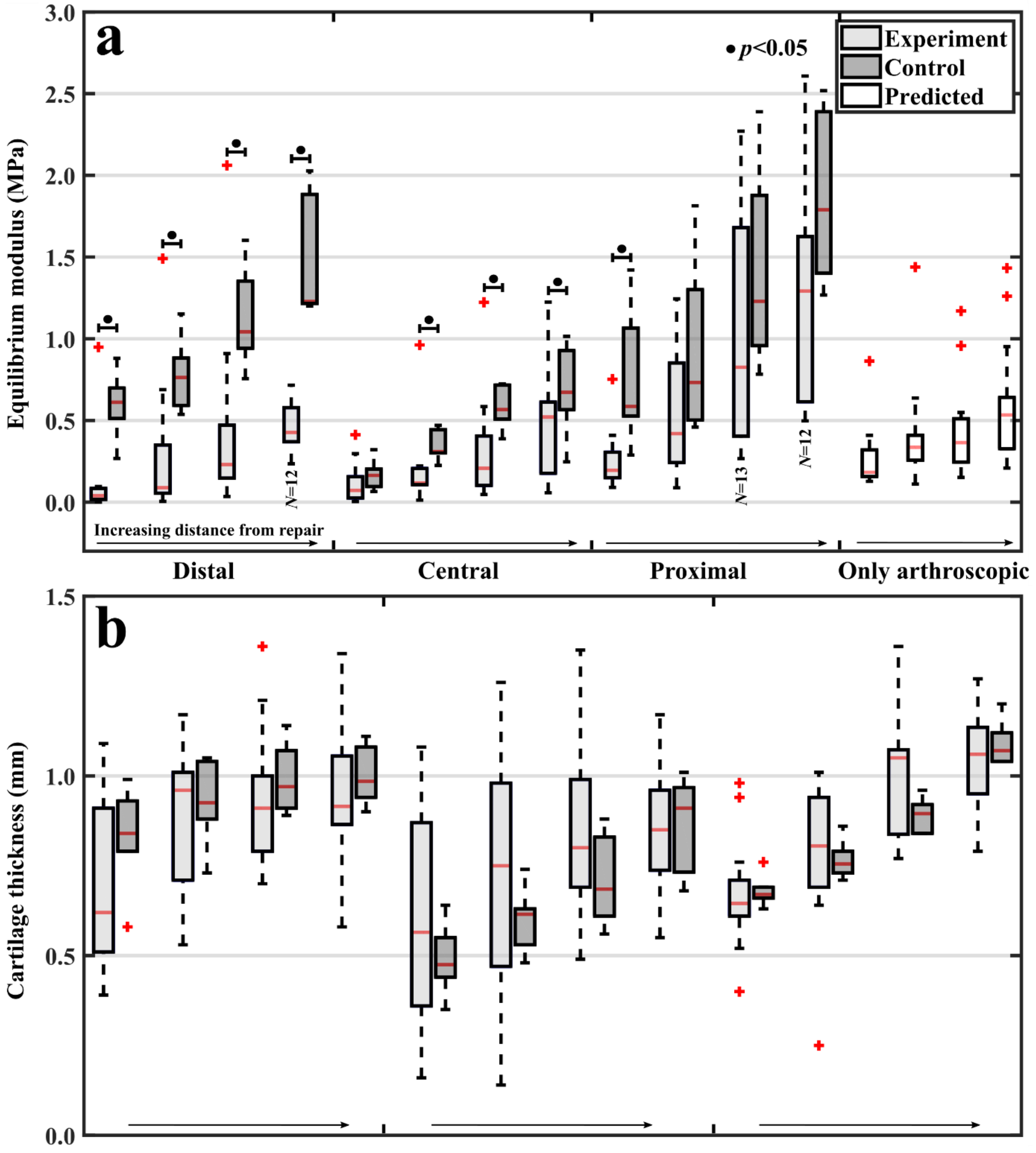

Supplementary Figure S1: Boxplots with median, quartiles (25% and 75%) and outliers of equilibrium modulus (**a**), and cartilage thickness (**b**) between the experimental and control groups. For each presented bar of experiment and control groups, the number of measurements were 14 and 6, respectively. Few locations had varying number of measurements (marked in subfigure **a**) due to limited accessibility during arthroscopy. Additionally, predictions from the locations with only arthroscopic NIRS measurements (Figure 2b, red dots) are presented (white bars). For each anatomical location (distal, central, and proximal), the leftmost bar presents the location closest to the repair.

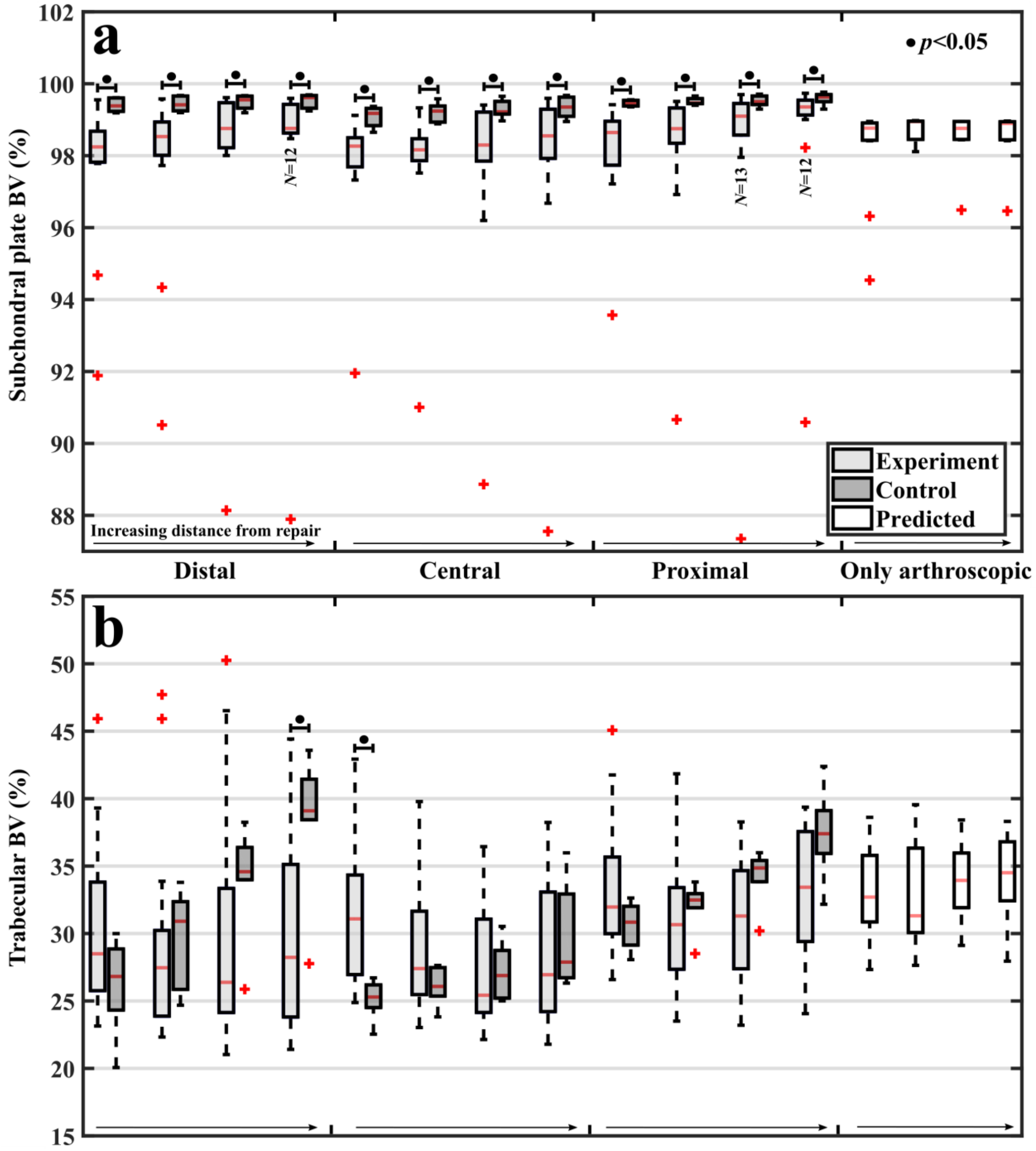

Supplementary Figure S2: Boxplots with median, quartiles (25% and 75%) and outliers of subchondral plate bone volume fraction (BV, **a**), and trabecular BV (**b**) between the experimental and control groups. For each presented bar of experiment and control groups, the number of measurements were 14 and 6, respectively. Few locations had varying number of measurements (marked in subfigure **a**) due to limited accessibility during arthroscopy. Additionally, predictions from the locations with only arthroscopic NIRS measurements (Figure 2b, red dots) are presented (white bars). For each anatomical location (distal, central, and proximal), the leftmost bar presents the location closest to the repair.

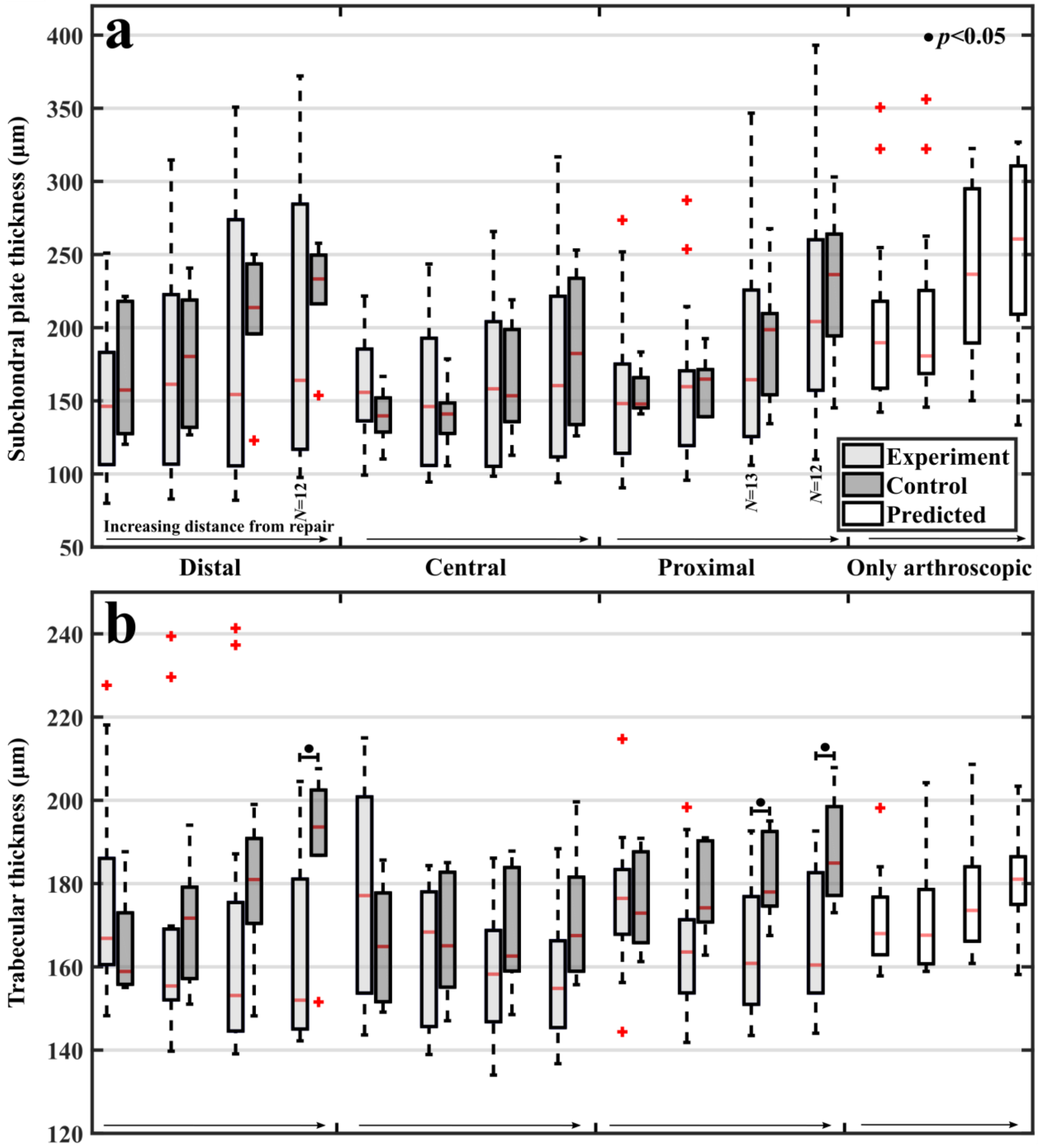

Supplementary Figure S3: Boxplots with median, quartiles (25% and 75%) and outliers of subchondral plate thickness (**a**), and trabecular thickness (**b**) between the experimental and control groups. For each presented bar of experiment and control groups, the number of measurements were 14 and 6, respectively. Few locations had varying number of measurements (marked in subfigure **a**) due to limited accessibility during arthroscopy. Additionally, predictions from the locations with only arthroscopic NIRS measurements (Figure 2b, red dots) are presented (white bars). For each anatomical location (distal, central, and proximal), the leftmost bar presents the location closest to the repair.

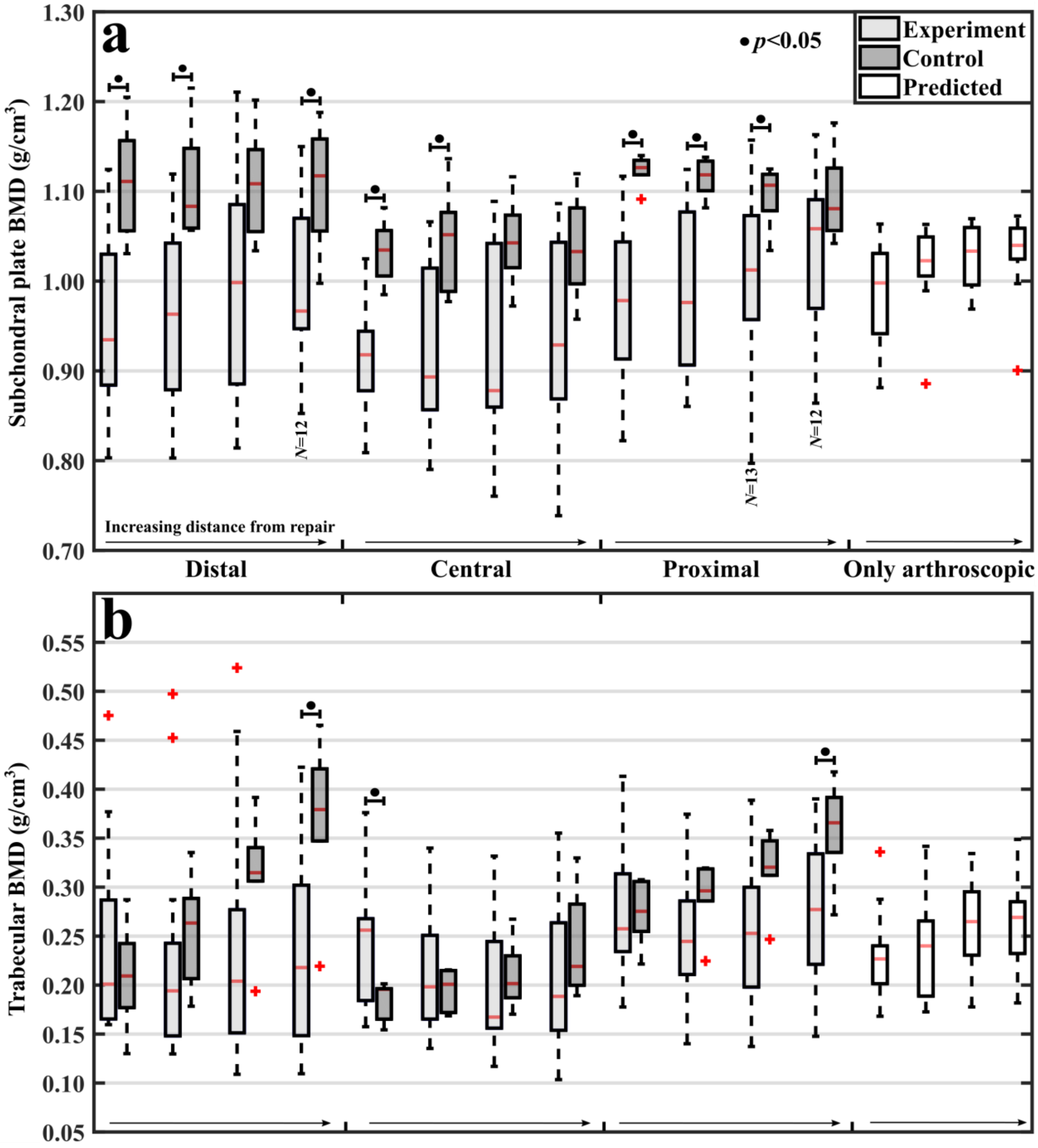

Supplementary Figure S4: Boxplots with median, quartiles (25% and 75%) and outliers of subchondral plate bone mineral density (BMD, **a**), and trabecular BMD (**b**) between the experimental and control groups. For each presented bar of experiment and control groups, the number of measurements were 14 and 6, respectively. Few locations had varying number of measurements (marked in subfigure **a**) due to limited accessibility during arthroscopy. Additionally, predictions from the locations with only arthroscopic NIRS measurements (Figure 2b, red dots) are presented (white bars). For each anatomical location (distal, central, and proximal), the leftmost bar presents the location closest to the repair.

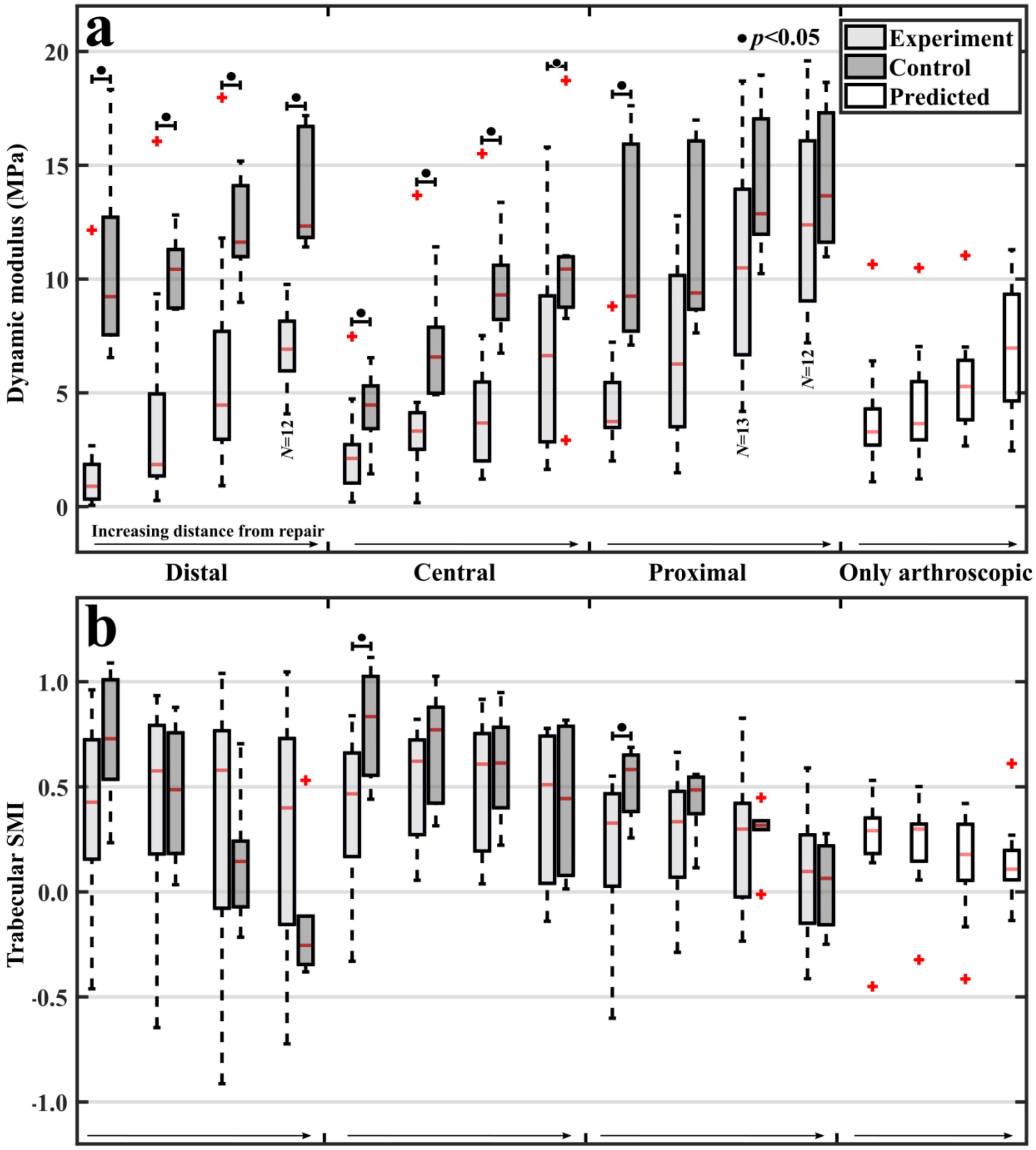

Supplementary Figure S5: Boxplots with median, quartiles (25% and 75%) and outliers of cartilage dynamic modulus (**a**), and trabecular structure model index (SMI, **b**) between the experimental and control groups. For each presented bar of experiment and control groups, the number of measurements were 14 and 6, respectively. Few locations had varying number of measurements (marked in subfigure **a**) due to limited accessibility during arthroscopy. Additionally, predictions from the locations with only arthroscopic NIRS measurements (Figure 2b, red dots) are presented (white bars). For each anatomical location (distal, central, and proximal), the leftmost bar presents the location closest to the repair.
